# Analysis of expressed sequence tags from *Prunus mume *flower and fruit and development of simple sequence repeat markers

**DOI:** 10.1186/1471-2156-11-66

**Published:** 2010-07-13

**Authors:** Xiaoying Li, Lingfei Shangguan, Changnian Song, Chen Wang, Zhihong Gao, Huaping Yu, Jinggui Fang

**Affiliations:** 1 College of Horticulture, Nanjing Agricultural University, Nanjing 210095, PR China

## Abstract

**Background:**

Expressed Sequence Tag (EST) has been a cost-effective tool in molecular biology and represents an abundant valuable resource for genome annotation, gene expression, and comparative genomics in plants.

**Results:**

In this study, we constructed a cDNA library of *Prunus mume *flower and fruit, sequenced 10,123 clones of the library, and obtained 8,656 expressed sequence tag (EST) sequences with high quality. The ESTs were assembled into 4,473 unigenes composed of 1,492 contigs and 2,981 singletons and that have been deposited in NCBI (accession IDs: GW868575 - GW873047), among which 1,294 unique ESTs were with known or putative functions. Furthermore, we found 1,233 putative simple sequence repeats (SSRs) in the *P. mume *unigene dataset. We randomly tested 42 pairs of PCR primers flanking potential SSRs, and 14 pairs were identified as true-to-type SSR loci and could amplify polymorphic bands from 20 individual plants of *P. mume*. We further used the 14 EST-SSR primer pairs to test the transferability on peach and plum. The result showed that nearly 89% of the primer pairs produced target PCR bands in the two species. A high level of marker polymorphism was observed in the plum species (65%) and low in the peach (46%), and the clustering analysis of the three species indicated that these SSR markers were useful in the evaluation of genetic relationships and diversity between and within the *Prunus *species.

**Conclusions:**

We have constructed the first cDNA library of *P. mume *flower and fruit, and our data provide sets of molecular biology resources for *P. mume *and other *Prunus *species. These resources will be useful for further study such as genome annotation, new gene discovery, gene functional analysis, molecular breeding, evolution and comparative genomics between *Prunus *species.

## Background

Mei (*Prunus mume*) originated in China and belongs to the sub-family Prunoideae within the Rosaceae family and is widely cultivated in East Asian countries. Mei trees are classified into two groups, fruiting mei and flowering mei, based on their uses [1]. The fruiting mei has been cultivated in China for more than 7000 years according to the historical records, and now it is also widely cultivated in Japan [2]. The processed products of mei include salted mei, mei wine and juice have high nutritional and medicinal value and are consumed in some countries, including China, Japan, and Korea. Chu [3] reported that mei flower and young fruit can tolerate relatively low temperatures (-4°C ~ -2°C) in early spring. These attributes make it an economically important and excellent ornamental tree in south China. Mei has long played an important role in human diet and health, but there is dearth of information about its flower and fruit development, physiology and biochemistry. The scarcity of knowledge contributes to difficulties in breeding, production, and storage of fruits. Mei cDNA library construction and EST analysis could be employed for the study of important genes responsible for flower and fruit development and their evolution in the plant kingdom.

Expressed sequence tags (ESTs) are partial sequences of expressed genes prepared by reverse transcribing mRNA and cloning the cDNA fragments into a plasmid and they are also gained by large scale sequencing at one instance [4]. They represent a snapshot of genes expressed in a given tissue and/or at a given developmental stage [5]. In plants, this method was initially applied to the model plant *Arabidopsis thaliana *[6]. Recently, many EST libraries of a wide range of plant species have been constructed for the genes involved in plant growth and differentiation [7], secondary metabolism [8] and biochemical pathways [9,10] as well as responses to pathogen attack and environmental stresses [11]. ESTs are proven cost-effective tools and represent abundant valuable resources for genome annotation, gene expression, and comparative genomics in non-model plants [12,13]. EST has also played an important role in functional genomics research on new functional gene discovery other than the whole genome [14-16]. By April 23, 2010, a total of 65,389,085 ESTs had been submitted to the National Center for Biotechnology Information (NCBI) from 1,989 species. The numbers of ESTs submitted are increasing rapidly at an approximate monthly rate of one million.

EST libraries for fruit trees such as grape, citrus, almond, strawberry, apricot, apple, blackberry and peach [12,13,17-22] have been constructed, sequenced, analyzed and deposited in databases. However, for *P. mume*, only 116 hits were found in the dbEST of the GenBank http://www.ncbi.nlm.nih.gov/dbEST/dbEST_summary.html in May 2010, a much lower figure than those of other main *Prunus *plants. In this study, a suitable high quality cDNA library from flowers and fruits of mei was constructed for the analysis of expressed sequence tags (ESTs), and to generate an EST resource for EST-SSR development of *P. mume*, the cDNA-based design of microarrays needed for understanding gene expression, and provide a platform for mei functional genomic studies on growth, development, metabolic regulation mechanisms, and so on. In this study, 1,294 unique sequences generated from 4,473 unigenes have homology with other plants, and 1,130 could be annotated with known and putative functions.

EST-SSR has been applied in molecular biology studies of fruiting trees as a new technology, especially on its application in some research fields of pomology, including genetic diversity analysis [23], genetic linkage map construction [24,25], comparative mapping [26,27], molecular phylogeny and cultivar identification [28]. Based on these EST resources, types and frequency of simple sequence repeats (EST-SSRs) with a motif length of 2-6 bp were searched by MISA software. The EST-SSRs generated in this study will be an efficient tool for additional genetic and genomic research in mei and even in *Prunus *species.

## Results

### Quality inspection of the cDNA library

The cDNA library of mei was constructed based on the pDNR-LIB vector (Invitrogen Biotechnology Co., Ltd). The results showed that the primary titer of the constructed cDNA library was 1.4 × 10^6 ^pfu/mL, while the recombination rate was about 97.5%, and the distribution of insert sizes of the library was about 1.0-3.0 kb based on random PCR analysis of 30 clones (Table 1). These results indicated that the constructed mei cDNA library was of a high quality (high titer, high recombinant rate and large inserted fragments) and it is also the first cDNA library of mei flower and fruit.

**Table 1:** Quality inspection and summaries of EST sequence analysis of the cDNA library of *P. mume*.

Group		Records
Primary titer		1.4 × 10^6 ^pfu/mL
Recombination rate		97.5%
Insert sizes		1.0-3.0 kb
Number of raw sequences		10,123
Number of high quality sequences		8,656
Average EST length		464 bp
Number of unigenes		4,473
Singletons		2,981
Contigs		1,492
Average unigene length		560 bp
Average number of ESTs per unigene		1.9
Average number of ESTs per contig		3.8
Maximum number of ESTs per contig		84
Minimum unigene length		101 bp
Maximum unigene length		1471 bp
Minimum ORF length		102 bp
Maximum ORF length		1323 bp
Average ORF length		383 bp

### ESTs obtained and assembled

A total of 10,123 clones were randomly picked and subjected to sequencing for the flower and fruit cDNA library. The leading vector, tailing of the sequence, and poor-quality sequences were excluded first, and 8,656 non-redundant EST sequences were obtained, showing a high success rate of sequencing. The average length of ESTs without vector was 464 bp. 4,473 non-redundant unigenes were assembled and most of them (3,672 unigenes, 82.1%) have sequence lengths between 400 and 800 bp, and only 2.4% (110) were shorter than 200 bp (see Additional file 1). The average length of unigene sequences was 560 bp, which was longer than that in other fruit trees such as persimmon [29], grapevine [12,30], apricot [20] and citrus [31], but shorter than almond [18] and peach [22]. The minimum and maximum length of these unigenes were 101 bp and 1,471 bp, and the average number of ESTs per unigene was 1.9. These unigenes were composed of 1,492 (33.36%) contigs and 2,981 (66.64%) singletons, with the number of average and maximum ESTs per contig being 3.8 and 84, respectively (Table 1). Only 11.6% of the contigs were composed of more than 4 ESTs (Additional file 2), suggesting that the redundancy rate is relatively low in this normalized library. In addition, the sequences data were submitted to the EST database of NCBI and publicly available (GW868575-GW873047).

### EST annotation and functional classification

We used BLASTX to annotate our *P. mume *unigene sequences. 1,294 (28.9%) of the unigenes matched genes in other species with an expectation value of 1e^-10 ^or better in a search against the NCBI nr protein database (released in October 2009), which is lower than other *Prunus *species, such as apricot [20], and peach [22]. That may because mei is different from other stone fruit species or the total number of ESTs sequenced were less than in the other two species, or for other reasons. The unigenes which showed similarity with sequences in public databases were classified into 23 groups based on their similarities as in Table 2. The results revealed that 1,130 (87.33%) of matched unigenes were known or predicted functional genes, 164 (12.67%) with unknown functional. Those unigenes with unknown functions could be considered as novel or specific genes in mei. Based on the known or predicted annotation, a large number of unigenes (353, 31.24%) were involved in transport and metabolism including carbohydrate, amino acid, lipid, inorganic, coenzyme and nucleotide. There were also 56 (4.95%) unigenes involved in DNA/RNA processing and transcription. Only 8 were seen to be involved in the defense mechanisms covering 0.62% of the functional genes.

**Table 2:** Grouping of unigenes from flower and fruit cDNA library of *P. mume*.

Category	Number of ESTs in Category	Percentage (%)
General function prediction only	237	18.32
Function unknown	164	12.67
Posttranslational modification, protein turnover, chaperones	146	11.28
Carbohydrate transport and metabolism	113	8.73
Amino acid transport and metabolism	95	7.34
Energy production and conversion	94	7.26
Translation, ribosomal structure and biogenesis	86	6.65
Lipid transport and metabolism	58	4.48
Inorganic ion transport and metabolism	42	3.25
Cell wall/membrane/envelope biogenesis	37	2.86
Coenzyme transport and metabolism	37	2.86
Signal transduction mechanisms	36	2.78
Replication, recombination and repair	26	2.01
Transcription	26	2.01
Secondary metabolites biosynthesis, transport and matabolism	25	1.93
Cytoskeleton	17	1.31
Intracellular trafficking, secretion, and vesicular transport	16	1.24
Cell cycle control, cell division, chromosome partitioning	16	1.24
Nucleotide transport and metabolism	8	0.62
Defense mechanisms	8	0.62
RNA processing and modification	4	0.31
Chromatin structure and dynamics	2	0.15
Cell motility	1	0.08
**Total**	**1294**	**100**

### Alignment against EST database of *Prunus *species

**Figure 1:**
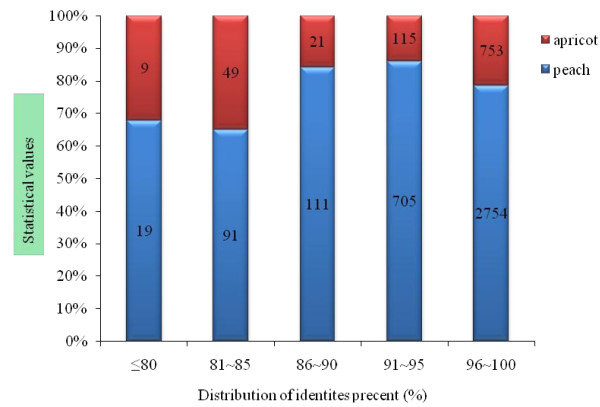
**Identities distribution of alignment results against the EST database of two other *Prunus *species**. The data in red and blue column were the numbers of mei ESTs which have homology with apricot and peach EST database in different identities.

By April 28, 2010, 100,071 ESTs from all *Prunus *species had been deposited in NCBI. However, only 116 ESTs were those of *P. mume *and this data was much lower than those *Prunus persica *(peach; 79,567) and *Prunus armeniaca *(apricot; 15,105). It is evident that the ESTs deposited from this work will provide vital data for *P. mume *genomic and genetic study. Here, we first carried out alignment between the unigenes (4,473) generated from *P. mume *and the EST database of two other *Prunus *species (*P. persica*, *P. armeniaca*) openly accessible in NCBI (by April 23, 2010). The results showed that 948 unigenes of mei aligned with *P. armeniaca*, at an identity level of 79%~100% (the average level was 96.2%), while 3,680 were aligned with *P. persica *and the average identity level was 96.53%. The numbers and distribution of identity level of mei unigenes which have aligned with two other species are shown in Figure 1. It was clearly discernible that most of the identity levels were over 96%, and 753 and 2,754 mei ESTs have homology with apricot and peach, respectively. The description above indicated that most of the *P. mume *ESTs had predicted functions and could be a great help for further research on functional genomics and genetic analysis of *P. mume*.

### Characterization of microsatellite sequences

Microsatellites or Simple Sequence Repeats (SSRs) have become one of the most useful molecular marker systems in plant breeding [32]. They are widely used in cultivar fingerprinting, genetic diversity assessment, molecular mapping, and marker assisted breeding. The development of SSR markers from genomic libraries is expensive and at times inefficient [33]. However, with the availability of large numbers of expressed sequence tags (ESTs), development of SSR markers from ESTs through data mining has become an efficient and low cost option for many plant species. In this study, MISA http://pgrc.ipk-gatersleben.de/misa was used to identify SSR markers in the unique sequences, and the results showed that 4,352 sequences were examined, with a total size of 2,455,352 bp, including 1,233 SSRs distributed in 935 sequences. 225 sequences were examined in more than 1 SSR loci and 179 SSR loci were present in compound formation. The SSRs found are summarized in Table 3. There were a total of 1,233 SSR sites comprising of 438 (35.52%) dinucleotides, 510 (41.36%) trinucleotides, 159 (12.9%) tetranucleotides, 53 (4.3%) pentanucleotides, and 73 (5.92%) hexanucleotides in the EST datasets. 41.36% (510) of these are the tri-nucleotide repeats (Table 3), which is in agreement with previous studies of other plant species [34-36]. The main type of dinucleotides was AG/CT with a frequency of 93.15% (408) and the second was AC/GT (16, 3.65%). The most frequent tri-nucleotide repeat was AAG/CTT (195, 38.24%) described in additional file 3.

**Table 3:** Distribution to different repeat type classes of EST library in mei.

Unit size	Number of SSRs	Percent (%)	Frequency (%)	Average distance(Kb)
2	438	35.52	10.06	5.61
3	510	41.36	11.72	4.81
4	159	12.9	3.65	15.44
5	53	4.3	1.22	46.33
6	73	5.92	1.68	33.63
**Total**	**1233**	**100**	**28.33**	**1.99**

**Figure 2:**
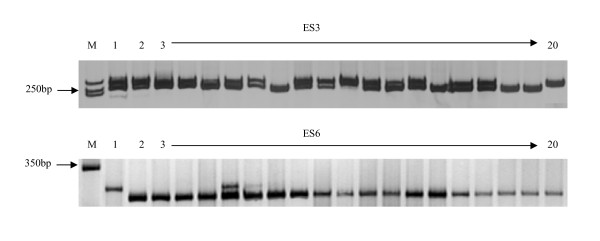
**Polyacrylamide gel electrophoresis patterns of microsatellite alleles amplified in *P. mume *using ES3 and ES6 SSR markers**. **M**: Maker DL2000; **1-20**: Accession numbers of *P. mume*, representing cultivar names were listed in Table 5.

To investigate whether the potential SSR loci mined are the true-to-type one and can be used for genetic analysis, 42 EST-SSR primer pairs were designed and verified in 20 mei cultivars. The PCR amplified results showed that only 14 (33.3%) could generate clear DNA banding patterns with the expected size. Two polyacrylamide gel electrophoresis patterns (amplified with ES3 and ES6 primers) are shown as examples in Figure 2. The information of these SSRs primers are shown in Table 4.

**Table 4:** Summaries of EST-SSR primers and repeat motif.

Primer	Repeat motif	**Primer sequence (5'**→3')*	Tm(°C)	Expected product (bp)
ES1	(AG)10	F: CTCTTGTTTCTCCTCCTACT	57.6	253
		R: GACGAGTTTATCGTTGACT		
ES2	(TG)13+(TC)20	F: TTGAGGTTGAGCCTACAC	56	306
		R: GCAAGGTCACTATCTTTTTC		
ES3	(TGG)7+(TGGTGT)3	F: GCTATACACGCTGCTAATA	54.6	259
		R: AAGGCATCACATCAACAC		
ES4	(AGC)7	F: CCGTAATAACCACCGTCC	58.2	240
		R: CCGCCTTCATCATCCTCT		
ES5	(CAA)8	F: CCAGATCCACTATTTCTTC	55.9	267
		R: GTGTTAGAGCCAGAAACC		
ES6	(AGTG)5	F: GCACTCTTCTCTCTCTCTCT	56.5	312
		R: GAGACCTTATGGAAGAAAAC		
ES7	(TTTTG)4	F: GAGAGAGACAAACAAGTGAA	55.5	253
		R: CTTGAGGAGTGATTTCCTA		
ES8	(TC)23	F: ACAGTTTCAGAATCTCACAG	57.6	284
		R: GATGGGACTTAAGAAGAGTC		
ES9	(AG)15	F: CCCTCTTATTCTCTCTCACT	56.5	207
		R: CTTCAATATACTTGGTGAGC		
ES10	(GA)22	F: CTACGTACTTCCTGAGTGAG	56.5	220
		R: CTAAAGATCGTTCAGACTGT		
ES11	(GTCT)3(GA)13	F: GGGTGTTGTGTCTGTTGGA	58.7	287
		R: ACGAGGAAGATGAGGAGGG		
ES12	(TTG)7	F: TTCCTGCTATCTGCTCCAAT	58.7	344
		R: GTGACGATGCTGTGCTCTGT		
ES13	(AT)10	F: GAGGAAATATTCCTGCATCA	55.5	188
		R: CTGTTTCGTCATCTTTTTCC		
ES14	(AGA)7(GGA)4	F: ACATATCCACCACCACCAAC	58.6	254
		R: AAAACAGAACACGACCCAGA		

* Every two primers belong to one pair. **F **is the forward and **R **is the reverse primer.

### Transferability of *P. mume *EST microsatellite markers

**Figure 3:**
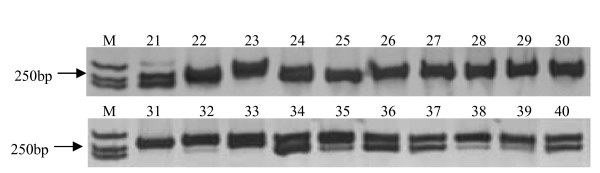
**Polyacrylamide gel images of SSR bands amplified in 20 cultivars of *P. persica *(peach) and *P. salicina *(plum) by primer pairs 3**. **M**: Maker DL2000; **21-30, 31-40**: Accession numbers of *P. persica and P. salicina*, the cultivar names were listed in Table 5.

The application of a PCR-based marker system for comparative genomics would be highly desirable, because such a marker system can increase the efficiency of transferring genetic information across species. This transferability can also make the EST-SSR markers have good potential application in comparative genomics between various plants and in their genetic analysis [34,37,38]. In this study, 14 EST-SSR markers developed from *P. mume *ESTs were used to test the transferability on other two main *Prunus *species (peach and plum). Clear DNA fingerprints of PCR amplification using mei SSR primer could be obtained, such as the polyacrylamide gels of SSR bands amplified by primer pairs 3 (Figure 3). The results showed that 13 of the primer pairs could produce anticipated simple sequence repeat (SSR) bands in plum and 12 in peach, and the rate of transferability was about 92% and 86%, respectively. This result indicated that the EST-SSR primers generated from mei had a higher generality among *Prunus *species.

**Table 5:** List of the cultivars used in this study.

**No**.	Cultivar name	Type	**No**.	Cultivar name	Type
1	'Koushuu Koume'	*P. mume*	21	'Shantao'	*P. persica*
2	'Zaohuamei'	*P. mume*	22	'Xuanchengtiantao'	*P. persica*
3	'Lv' e mei'	*P. mume*	23	'Maotao'	*P. persica*
4	'Qijiangxingmei'	*P. mume e*	24	'Qidongyoutao'	*P. persica*
5	'Shuangtaomei'	*P. mume*	25	'Huozhu'	*P. persica*
6	'Sichaunbaimei'	*P. mume*	26	'Taobadan'	*P. persica*
7	'Zhizhimei'	*P. mume*	27	'Xiaguang'	*P. persica*
8	'Dayezhugan'	*P. mume*	28	'Baifeng'	*P. persica*
9	'Gyokuei'	*P. mume*	29	'Xinjianghuangrou'	*P. persica*
10	'Wanhong'	*P. mume*	30	'Pingbeizi'	*P. persica*
11	'Hongmei'	*P. mume*	31	'Dalimei'	*P. salicina*
12	'Oushuku'	*P. mume*	32	'Dongbeili'	*P. salicina*
13	'Henghe'	*P. mume*	33	'Xiaosuli'	*P. salicina*
14	'Xianmimei'	*P. mume*	34	'Bulin'	*P. salicina*
15	'Xiao'ou gongfen'	*P. mume*	35	'Chuandaojiutian'	*P. salicina*
16	'Shinano Koume'	*P. mume*	36	'Dashizaosheng'	*P. salicina*
17	'Taoxingmei'	*P. mume*	37	'Aoli'	*P. salicina*
18	'Momei'	*P. mume*	38	'Xiangjiaoli'	*P. salicina*
19	'Koushuu Saisyou'	*P. mume*	39	'Gaixiandali'	*P. salicina*
20	'Gojirou'	*P. mume*	40	'Changli15'	*P. salicina*

Note: The detail characteristic of *P. mume *cultivars are described in China fruit records-Mei and reference 2; the detail characteristic of *P. persica *cultivars are described in China fruit records-Peach and *P. salicina *are described in China fruit records-Plum.

### Analysis of genetic diversity

**Figure 4:**
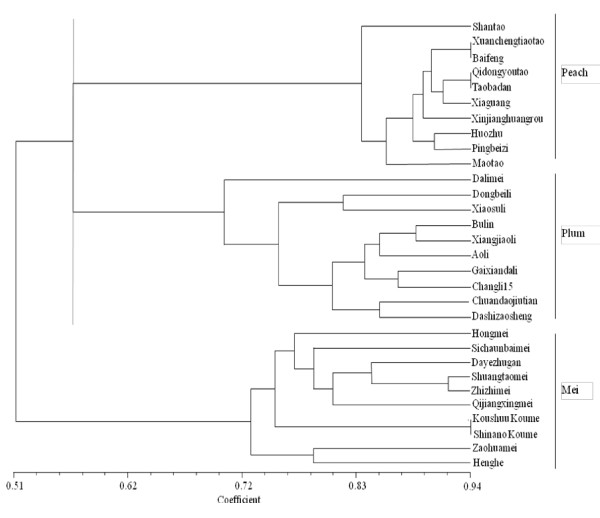
**A dendrogram constructed using polymorphisms at 14 SSR loci with a total of 54 alleles**. All these varieties of the three species were analyzed into three groups. The ten cultivars above were belong to *P. persica *marked as peach and middle were *P. salicina *marked as plum, down were *P. mume *marked as mei.

The genetic diversity among 30 varieties of peach, plum, and mei was analyzed with the new SSR primer pairs developed. 54 bands were totally amplified and high level of band polymorphism rate was observed in the mei species (70%), lower in plum (65%) and lest in peach (46%). A clustering diagram (Figure 4) showed that cultivars of these three species were divided into three groups, and the genetic difference among them was obvious, which was consistent with the fact that they are three different species. In the peach group, varieties belong to *P. persica *(L.) Batsch, such as 'Xuanchengtiantao' and 'Baifeng', 'Qidongyoutao' and 'Taobadan', 'Huozhu' and 'Pingbeizi' were clustered into three subgroups, with high similarity coefficients of 0.94, 0.94, 0.90, respectively. In the plum group, cultivars that had same geographical distribution were clustered together, such as 'Bulin' and 'Xiangjiaoli', with a genetic similarity coefficient of 0.88. In the mei group, all the ten cultivars were separated well, with 'Koushuu Koume' and 'Shinano Koume' introduced from Japan being clustered together. These results indicated that these *P. mume *SSR markers were useful in the evaluation of genetic relationships between and within the *Prunus *species.

## Discussion

Mei is an important fruit crop and famous ornamental tree in China, with processed mei products playing an important role in the economy and in people's daily lives. Expressed sequence tags (ESTs) are sequenced portions of messenger RNA, the projects of EST have been initiated for numerous plant and animal species, and generated large amount of sequence information that can be used for gene discovery, functional genetic studies, and marker development [39]. As we know, much more molecular researches have been carried out on mei, but the studies on flower and fruit development are poor. In this study, a high quality cDNA library of mei containing highly variable EST sequences was constructed, using a mixture of RNAs from plant materials at various stages, so as to understand the molecular mechanisms of growth, development and metabolic regulation at different development stages. The sequencing of mei ESTs can definitely provide an important platform for the studies of functional genomics, and can greatly accelerate the new gene discovery and study on EST-SSR development of *P. mume*.

In this study, the results of EST annotation and functional classification show that 1,130 unigenes have similarities to *Vitis vinifera*, *Populus trichocarpa *and *P. persica*. In the classification of known or putative functions (Additional file 4), the largest proportion of functionally assigned ESTs fell into three categories: primary transport and metabolism, protein synthesis and energy. Alignment of present ESTs was useful for comparative genomics. In this paper, we first compared the ESTs of *P. mume *with other *Prunus *plants, the results showed that these ESTs sequences were conserved also had high homology with other *Prunus*, which providing an important information for comparative genomics in *Prunus *species.

The scope of EST-SSR marker development is limited to the species for which sequence databases already exist. The SSR marker development for plant species lacking a sequence database can still be expensive and time consuming. An alternative approach for SSR marker development in those species could be the utilization of SSR markers from related species [32]. As the EST-SSR markers are derived from transcribed regions of DNA, they are expected to be more conserved and have a higher rate of transferability and polymorphism than genomic SSR markers [40]. By virtue of the sequence conservation of transcribed regions of the genome, a significant portion of the primer pairs designed from EST-SSRs is expected to function in distantly related species [36]. Transferability of EST-derived markers over different taxonomic levels has been demonstrated earlier [27,41]. In our study, the majority of our 14 EST-derived SSR loci from *P. mume *revealed cross-species amplification with alleles of comparable sizes in peach and plum. As expected, the transferability rate of the markers was high, which suggests that the flanking regions of these SSR loci are sufficiently conserved, and can be used for comparative analyses of genetic diversity, population structures and so on. It also indicated that these SSRs generated from *P. mume *can be useful for the development of conserved markers for linkage mapping and QTL analyses in different *Prunus *species.

In addition, the polymorphism rate in peach (46%) was lower than in plum (65%) and mei (70%). This may be due to the fact that most of the peach cultivars were from the same breeding program and thus have similar genetic background. The plum and mei cultivars have wider geographical distribution and bigger genetic differences. A dendrogram was constructed using the polymorphic bands in 30 cultivars of *P. mume*, *P. persica *and *P. salicina*, the relationships and diversity among these three species were analyzed accurately and can be roughly and visually reflected from the dendrogram. These results further suggest the applicability of mei EST-SSR markers in genetic study of *Prunus *species.

## Conclusion

This paper provides the first *P. mume *flower and fruit cDNA library. In addition, large scale and valuable EST information which can be great help to some further research about gene cloning, gene function and expression analysis of *P. mume *were generated. Our analysis further highlights the efficient identification of SSR from mei ESTs and the high transferability of mei EST-SSR markers between peach and plum.

## Methods

### Sample collection and DNA isolation

Fruits and flowers of *P. mume *'Xiyeqing' at different development stages were collected from the fruiting mei resource nursery of Nanjing Agricultural University, Najing PRC. The materials were collected from February 23, 2009 to May 6, 2009 and the collected tissues were frozen immediately in liquid nitrogen and stored at -40°C awaiting RNA extraction. The flowers were collected at four growing stages including small bud, medium bud, half-unfolding flower and completely unfolding flower, while the fruits were collected for seven weeks until the stone hardening stage, with the average size of 0.3, 0.6, 0.9, 1.2, 1.8, 2.6, 3.6 cm diameter.

All the samples of *P. mume *consisting of six introduced from Japan and used for EST-SSR analysis were collected on April 4, 2009 in this resource nursery and their names are described in Table 5. Cultivars of *P. persica *and *P. salicina *were obtained from National Germplasm Resources Garden of Peach in Jiangsu Academy of Agricultural Sciences, Najing PRC, the name of cultivars are also listed in Table 5. Genomic DNA was isolated from leaves using the DNeasy Plant Mini Kit (Qiagen, City, CA, USA) according to the manufacturer's recommendations.

### cDNA library construction

Total RNA from the selected flowers and fruits of each stage was extracted by using Trizol (Takara Biotechnology, Dalian Co. Ltd.) respectively, and then 1 μg from each stage were mixed, while cDNA library construction was carried out according to the Creator SMART cDNA Construction Kit protocol (Clontech, USA, Cat.No.634903). The quantity and integrity of RNA was detected by BioPhotometer (Eppendorf, Gene Company Limited) while electrophoresis was done on agarose with Ethidium Bromide. The first strand and double strand cDNA were synthesized according to the protocol of Creator SMART cDNA Construction Kit (Clontech, USA, Cat.No.634903).

For first strand cDNA synthesis: 3 μl RNA sample, 1 μl SMART IV oligonucleotide (5'-AAGCAGTGGTATCAACGCAGAGTGGCCATTACGGCCGGG-3') and 1 μl CDS-3 M adapter (10 μM, 5'-AAGCAGTGGTATCAACGCAGAGTGGCCGAGGCGGCC (T)_20_VN-3') were mixed and incubated at 72°C for 2 min and placed in ice for another 2 min. 2.0 μl 5 × First-Strand Buffer, 1.0 μl DDT (20 mM), 1.0 μl dNTP Mix (10 mM), 1.0 μl PowerScript™ Reverse Transcriptase (Invitrogen Biotechnology Co., Ltd) were added to a total volume of 10 μl, mixed well and incubated at 42°C for 1 hour.

Double strand cDNA synthesis: The single strand cDNA was amplified for double strand cDNA synthesis by long-distance polymerase chain reaction (LD-PCR), in the following steps, 2 μl single-strand cDNA were added to 80 μl ddH_2_O, 10 μl 10 × Advantage 2 PCR Buffer, 2 μl 50 × dNTP Mix, 4 μl M1 PCR Primer (10 μM, 5'-AAGCAGTGGTATCAACGCAGAGT-3'), and 2 μl 50 × Advantage 2 Polymerase Mix then topped with ddH_2_O to make a total volume of 100 μl. The mixture was centrifuged then amplified as follows: 95°C for 1 min; 20 cycles of 95°C for 15 sec and 68°C for 6 min. Five microliters of the centrifuged PCR product were pipetted onto a 1.2% agarose/EtBr gel to check the double strand (ds)-cDNA quality. The concentration of the ds-cDNA was estimated by comparing with a suitable DNA marker.

The remaining double-strand cDNA was digested by SfiI Enzyme then sized, purified by a QIA quick PCR Purification Kit (Qiagen Cat.No.2810) and packaged in order to filter the fragments shorter than 400 bp. Double strand cDNA with cohesive ends was finally ligated into pDNR-LIB vector by T4 ligase, the recombinant vector was transformed into *Escherichia coli *DH10B by electroporation at 2.1 KV, cultured overnight in 37°C after directly applying onto a media plate with chloramphenicol.

### Sequencing for the clones

After the cDNA library was plated onto LB media plates, the white clones were picked into a 96-well block containing 800 μl LB culture media (supplied with chloramphenicol 50 mg/ml) and incubated at 230 rpm and 37°C overnight. Before large scale sequencing, PCR reaction was initially conducted to check the size of inserted fragments using random selection of 30 clones [42]. Finally, about 10,000 fresh clones were sent to Beijing Huada Gene Company (Beijing, P.R. China) for sequencing.

### EST processing

Among the vector sequences, low quality and redundant sequences were rejected with Chromas, Phred [43] and phd2fasta. Sequences longer than 100 bp were outputted after converting to FASTA forms. Uniform sequences were assembled using CAP3 [44] software for splicing into contigs with two or more ESTs.

### EST annotation and function

For further functional annotation, comparative and classification analysis, the non-redundant sequences (edited ESTs sequences) considered as valid were subjected to BlastX analysis against the non-redundant protein database (nr) of National Center of Biotechnology Institute (NCBI) to search for similarity. Search results were imported into Microsoft Office Excel where sequence matches with E-value scores ≤10^-10 ^were considered significant and used to categorize the ESTs based on their putative or known functions of plant genes.

### EST alignment against *Prunus *EST database

The software package of blast was download from the FTP server ftp://ftp.ncbi.nlm.nih.gov/blast/executables/ of NCBI, then compiled and installed according to the instructions [45]. Alignment of nucleic acids were carried on among the ESTs of *P. persica*, *P. armeniaca *submitted in NCBI (by the end of April 1, 2010) and unigenes generated from *P.mume *cDNA library by using blastn with e^-10 ^as the parameter.

### Identification of EST-SSRs

The EST library was searched for sequences containing SSRs using MISA [37], a Perl script able to detect perfect as well as compound microsatellites in nucleotide sequences. Compound microsatellites were defined as repeats interrupted by a non-repetitive sequence of a maximum 100 nucleotides. MISA was set with the following minimum length criteria for the extraction of repeated units (unit size/minimum number of repeats): at least six dinucleotides (2/6); at least five trinucleotides (3/5); three tetranucleotides (4/3), three pentanucleotides (5/3) and three hexanucleotides (6/3). Sequences contained corresponding repeat units were selected for marker development.

### EST-SSR primers designed and PCR amplication

Primer pairs flanking repeats with a minimum length 20 bp were designed using by Primer3.0 program http://frodo.wi.mit.edu/primer3/ and the parameters are as follows: Primer GC%: 40% ~ 60%, and the optimum value of 50%; Primer size: 18 ~ 22 bp; Primer TM: 50 ~ 60°C; Product size ranges: 150-350 bp. The primers designed were also identified by Oligo6 software in order to avoid the primer dimers, hairpin structure and the occurrence of mismatch and so on. Primers were synthesized by Invitrogen, Shanghai PR China.

The PCR amplification reactions were performed using an Eppendorf Authorized Thermal Cycler each and conducted in a total volume of 25 μl containing 50 -100 ng template DNA, 20 pmol each primer, 2 Units Taq DNA polymerase (purchased from Takara Biotechnology, Dalian Co. Ltd.), 2.5 μl 10 × PCR buffer, 2 μl MgCl_2 _(2.5 mM), 1.5 μl dNTPs (2.5 mM), and added ddH_2_O to the total volume. The PCR conditions were: 94°C for 5 min, followed by 35 cycles of 94°C for 30 sec, 50 ~ 60°C for 40 sec, 72°C for 1 min, and a final extension step at 72°C for 10 min. To check for PCR product quality, 8 μl of the PCR reaction was visualized on a 1.5% agarose gel and the remaining reaction was then electrophoresis on 5% polyacrylamide gel in 1 × TBE buffer at 80 W for 100 to 120 min. Gels were stained with silver nitrate following the protocol detailed by Bassam [46] with few modifications and photographed in white light.

### Cross-species amplification

To assess the transferability of our EST-SSR markers, we tested their amplification in other *Prunus *species containing 10 cultivars of *P. persica *(peach) and 10 of *P. salicina *(plum). The PCR amplification reactions were performed as the same as above.

### Data Analysis

According to presence or absence of bands on the polyacrylamide gel, the same electrophoretic bands were recorded as 1, no bands were recorded as 0. The polymorphisms data were inputted into Excel for the cluster analysis using NYSYS-pc software and a dendrogram was generated for the analysis of genetic relationship and diversity among the varieties of peach, plum and mei.

## Authors' contributions

LX carried out the laboratory work and participated in manuscript draft writing. SL performed data analyses. SC, WC, GZ, and YH participated in the design and coordination the study. FJ conceived, designed the study and revised this paper. All authors read and approved the final manuscript.

## Supplementary Material

Additional file 1 **Length distribution of uineges in the flower and fruit cDNA library of fruiting mei**. This graph shows the description of the length distribution of uineges in the flower and fruit cDNA library of fruiting mei. Click here for file

Additional file 2 **Composition of contigs**. This table shows the composition of contigs in this study. Click here for file

Additional file 3 **Types and frequency of SSRs in this study**. Tables show the details of classified repeat types (considering sequence complementary). Click here for file

Additional file 4 **Classification of *P. mume *unigenes with known or putative functions**. This graph shows the detail classification of *P. mume uni*genes with known or putative functions. Click here for file
