# Supplementary material for: Analysis of expressed sequence tags from Prunus mume flower and fruit and development of simple sequence repeat markers
Source: BMC Genet. 2010 Jul 13;11:66. doi: 10.1186/1471-2156-11-66 (PMC2920227; doi:10.1186/1471-2156-11-66)
Supplement: Additional file 4 — Classification of P. mume unigenes with known or putative functions. This graph shows the detail classification of P. mume unigenes with known or putative functions. [file 1471-2156-11-66-S4.DOC]

**Additional file 4**

**Classification of *Prunus mume uni*genes with known or putative functions**
